# How far is single‐cell sequencing from clinical application?

**DOI:** 10.1002/ctm2.117

**Published:** 2020-07-05

**Authors:** Jiaqiang Zhang, William Wang, Jianan Huang, Xiangdong Wang, Yiming Zeng

**Affiliations:** ^1^ Department of Anesthesiology and Perioperative Medicine Center for Clinical Single Cell Biomedicine Henan Provincial People's Hospital, People's Hospital of Zhengzhou University Zhengzhou Henan China; ^2^ Center for Tumor Diagnosis and Therapy Zhongshan Hospital Institute of Clinical Science Fudan University Shanghai Medical College Jinshan Hospital, Fudan University Shanghai China; ^3^ Department of Pulmonary and Critical Care Medicine Clinical Center for Molecular Diagnosis and Therapy The Second Affiliated Hospital of Fujian Medical University Quanzhou Fujian Province China

**Keywords:** clinical application, single‐cell sequencing

Single‐cell sequencing (SC‐seq) is rapidly becoming one of the most important approaches to single‐cell biomedicine and is centered around single‐cell RNA sequencing (scRNA‐seq) and single‐cell DNA sequencing (scDNA‐seq). Growing evidence suggests that SC‐seq can significantly speed up the identification of new cell types, intercellular heterogeneity, function‐based sub‐categories, intelligent cell and human cell altars, disease biomarkers, therapeutic targets, and it can evaluate intra‐ and intercellular communication.^1^, ^2^, ^3^ Molecular characteristics of single cells are uncovered through transcriptome to chromatin accessibility, DNA mutation to histone modification, genome sequences to multi‐dimensions, phenomes to functions, and from dynamics to spatial alternations. SC‐seq is an approach that contributes to the understanding of interactions and communications between RNA‐DNA‐proteins, proteins‐proteins, cells‐cells, and pathogens‐host cells from which molecular mechanisms of disease pathogeneses and progression can be explored. Tumor heterogeneity is a critical factor that influences the response of tumor cells to chemotherapy and target‐based individualized therapy. New clinical strategies for precision medicine are designed according to tumor heterogeneity and dynamic mutations to improve the prognosis of patients with multiple metastases and reoccurrences.^4^ The value of SC‐seq in clinical and translational medicine lies in its ability to develop our understanding in tumor heterogeneity, genesis, evolution, and metastasis in various diseases.^5^, ^6^, ^7^, ^8^ SC‐seq also plays an important role in monitoring on/off‐targets and the post‐operation of gene editing for gene therapy.^9^ The aim of this editorial is to provide a brief overview of the recent development of scRNA‐seq, scDNA‐seq, single cell multi‐omics (scMO), trans‐omics (scTO), and to highlight the challenges that need to be faced during the clinical application of SC‐seq.

scRNA‐seq is no longer a foreign word for clinicians and is almost ready for clinical application. Single‐cell transcriptome profiles have been widely mapped in many human tissues/organs and diseases, due to the application of scRNA‐seq. Most studies on single cells from patients are designed to explore molecular mechanisms of diseases, map transcriptomic phenomes of disorders, uncover rare cell types and cellular subpopulations developed during disease occurrence, define new categories of disease and patient stratification, and to monitor therapeutic responses. Differences in single‐cell transcriptomic profiles are usually observed by comparing healthy patients to patients with disease. However, there is usually a lack in comparison based on clinical considerations, for example, among disease subtypes, stages, severities, durations, and dynamic responses to drugs. A key challenge is to develop a definition for scRNA‐seq platforming that can be used for clinical application, translation of single‐cell transcriptome phenomes to clinical precision medicine, and the dynamic monitoring of target cell‐specific responses to drugs. The integration of scRNA‐seq profiles with scDNA‐seq and genomic or proteomic phenomes has recently been proposed as it can aid in understanding transcriptome‐associated regulations and functions, heterogeneity‐dependent tumorigenesis, and the interaction among intracellular signal pathways, although comprehensive analyses needs to be standardized and uncomplicated.

Copy numbers, mutations, and modifications at a single cell resolution can demonstrate intercellular variations, metastatic lineages, and clonal lineages in cancer. scDNA‐seq is an approach that can help clinicians in understanding the existence and formation of aneuploidy evolution, phylogenetic trees, heterogeneity, and can prospectively predict the occurrence of metastasis while improving the quality of clinical precision therapy. Target gene panels have been developed for both single‐cell and bulk tissue DNA sequencing to improve on efficiency. Leung et al. modeled the clonal evolution and metastatic dissemination of colonic cancer in patients using scDNA‐seq, exome sequencing, and targeted deep‐sequencing and found the association between cancer cell evolution, acquired mutations numbers, and copy numbers with distant organ sites of metastasis.^10^ In contrast to tumor bulk sequencing, scDNA‐seq analyses could define the self‐seeding of tumor cells from the early‐dissemination of metastasis and discover complex clonal evolution within tumors. With the improvement of high‐throughput capacity, scDNA‐seq can be routinely and efficiently performed to detect tumor heterogeneity and evolution. In addition, scDNA‐seq is a critical approach to measure epigenetic modifications, for example, aberrant DNA and histone, DNA methylation, acetylation, phosphorylation, aberrant gene expression, imprinting loss, and chromosomal and microsatellite instability. Bohrson et al recently developed a new method named “linked‐read analysis” that can precisely profile somatic single nucleotide variants using read‐level phasing with nearby germline heterozygous polymorphisms and characterize mutational profiles while predicting somatic mutation rates in single cell level.^11^ This analysis resulted in more accurate sensitivity for single‐cell somatic heterozygous single nucleotide variants, especially for singletons and in the condition with high false discovery rates, while only can detect the part of the single‐cell genome that rounds polymorphic germline heterozygous single nucleotide variants. This method provides a new approach that can precisely link the number of mutations with age, mutational processes, and predict mutagenetic rates and intercellular heterogeneity.

scMO is a new alternative in clinical and translational medicine that provides multi‐dimensional insights into intracellular signal networks and functions and can help to define new function categories using comprehensive analyses. Wang recently described multi‐omics as the parallel association and correction among multiple molecular omic profiles.^12^ SC‐seq of genomes, DNA mutations, methylomes, and transcriptomes can demonstrate accurate mechanisms and how multiple network layers regulate and correlate among themselves. For example, some genomic copy‐number variations altered RNA expression of genes within the gained or lost genomic regions, rather than DNA methylation to address the association among genetic, epigenetic, and transcriptomic heterogeneities.^13^ The simultaneous and comprehensive analyses of single cell transcriptome, genome, epigenome, and proteome profiles can demonstrate genotypic and phenotypic characteristics of single cells at multiple layers and integrate regulatory mechanisms of cell evolution with their function. A new method of simultaneous DNA methylome and RNA transcriptome profiles in cells could detect the DNA methylation status of 0.5‐1 million CpG sites, mRNA expression of 10 000 genes, and both promoter DNA methylation and RNA transcription of one‐third genes.^14^ It is possible that the numbers of measured genes and methylation sites increase with the improvement of methodology.

scMO‐seq could play a crucial role in deepening our understanding of molecular mechanisms and the balance between heterogeneity and homogeneity of epigenomic reprogramming in reproduction and development. For example, the reprogramming of genome‐scale chromatin statuses and DNA methylation dynamics at the single‐cell stage of early embryos was evidenced by chromatin state/nucleosome positioning. DNA methylation, copy number variation, and ploidy were achieved using single‐cell multi‐omics sequencing technology. Single‐cell chromatins overall omic‐scale landscape sequencing was used to map DNA methylation, chromatin accessibility, and to find feedback mechanisms between transcription and open chromatin maintenance. Epigenetic reprogramming during human preimplantation development, that is, the inhibition of RNA transcriptional profiles leads to the closure of more than one‐third of the open chromatin regions in promoters of human early embryos.^15^ Early cell‐fate choice and the coordination among the three primary germ layers can be regulated by epigenetic reprogramming accompanied by alternations of transcriptional profiles during the onset gastrulation of embryos.^16^ The ten‐eleven translocation‐mediated demethylation and elevated chromatin accessibility could drive epigenome rearrangements, contributing to epigenetic prime or the remodeling of regulatory elements associated with each germ layer before cell‐fate decisions.

There is no doubt that scRNA‐seq, scDNA‐seq, and scMO/TO are important approaches to exploring the molecular mechanisms of transcriptional function, heterogeneity, epigenetic modification, chromatin inaccessibility, multi‐dimensional regulation, and control at single‐cell level. Single‐cell biomedicine is a new emerging discipline that will improve the understanding of molecular pathogenesis and pathophysiology, facilitate the discovery and validation of biomarkers and targets, strategize target‐based precision medicine, and positively impact the life quality of patients. The questions are whether single‐cell measurements are ready for clinical application and how far SC‐seq is from disease diagnosis, dynamical monitoring, therapeutic efficacy, and prognostic prediction. Luecken and Theis recommended a best‐practice of scRNA‐seq workflow in data generation and analysis, for example, how to preprocess and visualize raw data, perform quality control, normalize and standardize single cell gene expression and epigenetic modification, correct and integrate transcriptomic profiles with other omics, select and carry out multiple analyses and annotations, and interrupt results to clinicians and patients.^17^ With the maturation and development of SC‐seq methodologies, it seems that the concept of single‐cell biomedicine is becoming more accepted by clinicians and patients and the clinical translation of scRNA‐seq into real life practice needs to be further considered. It also needs to be further standardized and consortiums of clinical SC‐seq usage are urgently required. Lähnemann et al highlighted major challenges that existed in the translational process from single‐cell biology to clinical utilities, including sparsity, flexible statistical frameworks, single cell alters, trajectories, patterns in spatially resolved measurements, errors, and missing data, phylogenetic models, data and types of measurement, population genetic parameters, and analysis tools.^18^ For clinical practice, it is important to standardize the processing of tissue sampling from operations (eg, human sample resection, selection, transfer, and isolation from tissue to single cells), establishing criteria for scRNA‐seq, scDNA‐seq, and scMO/TO for each disease (eg, disease nature, severity, stage, duration, location, complexity, control pair, genetics, and history), and to simplify and minimize the procedures for measurements and analyses (eg, protocols for each disease and tissue, methodologies of data analyses, and clinical descriptions of result reports to clinicians). Of those factors, the most important issue is to clarify what scRNA‐seq, scDNA‐seq, and scMO/TO can provide for decision‐making, disease‐monitoring, and therapy‐assistance in clinical practice.

In conclusion, the fast development of scRNA‐seq, scDNA‐seq, and scMO/TO provides a new insight into intra‐ and intercellular communications and network interactions, and is a new emerging area that can uncover disease‐specific biomarkers and target‐based precision therapies. The integration of transcriptomic profiles, epigenetic modifications, and multi‐dimensional networks at single‐cell levels with clinical phenomes has a significant impact on the development of single‐cell biomedicine and will be a new strategy to discover new generations of diagnosis and treatment. Clinical translation of single cell measurements into routine practice is highly dependent upon the development of individual protocols and consortiums for each disease, tissue, and cell, with many challenges remaining.
